# Contribution of Indoor Air and Dust to Children’s Per- and Polyfluoroalkyl Substance Exposure in the Faroe Islands

**DOI:** 10.1021/acs.est.6c02003

**Published:** 2026-07-30

**Authors:** Melissa Woodward, Jitka Becanova, Simon Vojta, Pál Weihe, Philippe Grandjean, Rainer Lohmann

**Affiliations:** † 54083 University of Rhode Island Graduate School of Oceanography, Narragansett, Rhode Island 02882, United States; ‡ Department of Occupational Medicine and Public Health, Sigmundargøta 5, Tórshavn FO-100, Faroe Islands; § National Institute of Public Health, 195024 University of Southern Denmark, Studiestræde 6, Copenhagen 1455, Denmark; ∥ Department of Biomedical and Pharmaceutical Sciences, University of Rhode Island, Kingston, Rhode Island 02881, United States

**Keywords:** PFAS, Human exposure, Remote regions, Atmospheric sampling, Sampling tools

## Abstract

The people of the Faroe Islands are uniquely exposed to per- and polyfluoroalkyl substances (PFASs); despite no local production of these compounds or manufacturing of consumer products, residents exhibit elevated serum concentrations of PFASs. Consumption of pilot whales, a traditional part of the Faroese diet, contributes to exposure but does not fully account for observed trends in serum, suggesting the importance of additional exposure pathways. This research assessed the significance of indoor exposure pathways through measuring air and dust concentrations of a broad scope of PFAS nonvolatile precursors, volatile neutral precursors, and legacy ionic PFASs, and determining the estimated daily intake (EDI) for 15-year-old children. Across the 40 homes, the EDI for children from indoor exposure was 0.001, 0.012, and 0.129 ng/kg bw for low, intermediate, and high exposure scenarios, respectively. Indirect exposure to perfluoroalkyl carboxylic acids from air contributed the most to the EDI, followed by indirect exposure to perfluorooctanesulfonic acid in dust in the high exposure scenario. Indoor exposure equated to approximately 0.2% (low), 2% (intermediate), and 20% (high) of the European Food Safety Authority (EFSA) tolerable weekly intake, while the average consumption of pilot whale contributed about 60%. This study highlights the importance of understanding indoor exposure as a potentially significant contributor to total exposure to PFAS, especially with the declining consumption of pilot whales in the Faroese population.

## Graphical abstract

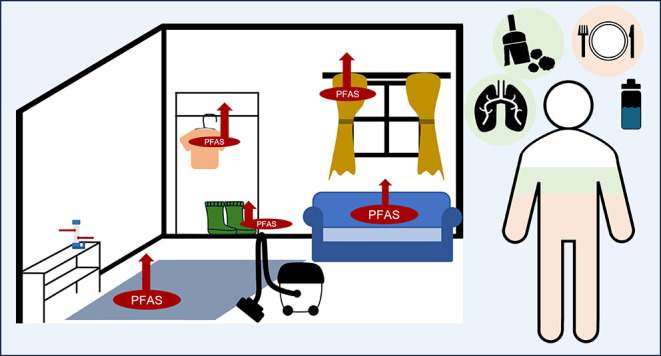

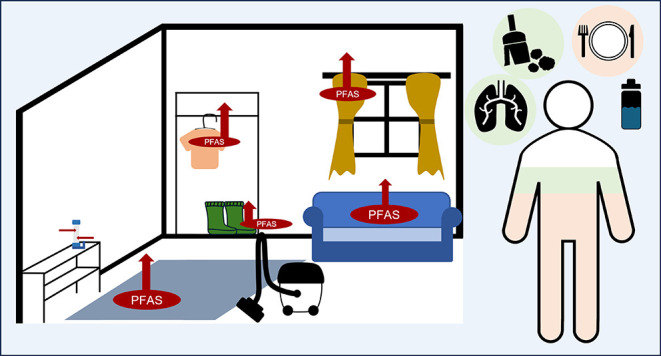

## Introduction

Since the 1950s, per- and polyfluoroalkyl substances (PFASs) have been heavily utilized in a wide array of industrial processes and consumer products due to their ability to enhance materials with stain-resistant and waterproof properties.^1–3^ Such properties make them ideal for use in applications such as water- and grease-proof coatings, textile treatments, food packaging, and surfactants, but PFASs have been shown to cause adverse health effects in humans.^4,5^ The presence and use of such consumer products within homes and the fact that humans typically spend 90% of their time indoors^6^ mean that inhalation of indoor air and ingestion of dust could be important pathways for human exposure to PFASs.^7^

Most previous studies have focused on PFAS occurrence in residential environments in regions neighboring locations where PFASs and/or consumer products are manufactured.^8–18^ However, despite our knowledge that PFASs are globally dispersed, little research has been done to assess their presence in communities far from PFAS production and application sites.^19–21^ Furthermore, measurements of air concentrations within residential homes are limited due to the lack of a widely accepted, reliable, and easy tool to detect PFASs in air.

Atmospheric measurements of PFASs are typically conducted using either active or passive sampling. Active sampling often relies on large, electricity-dependent equipment, which limits its suitability for homes or studies requiring extensive spatial coverage.^22,23^ Alternatively, the use of passive sampling provides an easier and cheaper method of measuring PFASs in the air.^24,25^ In a recent study, a polypropylene radial diffusive tube filled with XAD sorbent was deployed alongside polyethylene (PE) sheets in a clothing warehouse.^26^ The sorbent-filled tube detected similar concentrations of the target neutral PFASs measured, for example, fluorotelomer alcohols (FTOHs), as the PE sheets.^26^ This pilot study demonstrated handling ease of the passive sampler, showing promise as a cost-effective tool for the detection of atmospheric PFASs, especially in home environments.

The Faroe Islands consist of around 12 inhabited islands with a population of approximately 55,000. They are located in the North Atlantic Ocean, northwest of Scotland, east of Iceland, and west of Norway. The Faroe Islands are a self-governing nation under the external sovereignty of the Kingdom of Denmark. The islands have a prosperous fishing community and maintain traditional dietary practices including the consumption of pilot whale meat and blubber. The population is considered highly homogeneous with a long history of shared culture and ancestry. Homes across the Faroe Islands are also relatively uniform, with many sharing similar styles and materials.

In the Faroese community, elevated pollutant concentrations, including PFASs, have been measured in humans, despite the lack of production of PFASs or manufacturing of consumer products.^27^ This has been linked to the consumption of pilot whales as part of their traditional diet for centuries. Although consumption has declined over recent decades following dietary recommendations, pilot whales remain a significant source of PFAS exposure in this location far from major pollution sources.^28^ Previous work by Dassuncao et al., 2018, reported that while pilot whale consumption contributes to PFAS exposure in the Faroese population, it does not explain the observed trends in serum PFAS concentrations, suggesting that additional exposure pathways are also important.^28^

Starting in 1985, cohort studies have been conducted on the Faroe Islands, initially established to study the effects of mercury but since have expanded to include PFASs.^29–31^ Research from these cohort studies was the first to identify immune suppression, specifically reduced vaccine response, as one of the key effects of PFAS exposure.^30,31^ We leveraged the fifth birth cohort (cohort 5), for which serum PFAS concentrations were determined at age 15 years,^31,32^ to explore sources other than diet to better understand the total exposure and identify the dominant exposure pathways in the community. This cohort recruited participants from subsequent childbirths during 2007–2009, with PFAS concentrations first assessed in serum from cord blood collected at birth and subsequently measured in serum about every 5 years.^31^

The main objectives of this research were (i) to measure indoor air concentrations and dust concentrations of PFASs in homes across the Faroe Islands, (ii) to demonstrate the use of air–dust partitioning to predict concentrations in indoor matrices, (iii) to estimate daily intake of PFASs via air inhalation and dust ingestion and compare it to other exposure pathways, and (iv) to identify any significant relationship between indoor concentrations of PFAS and concentrations measured in humans.

To assess the significance of air inhalation as an exposure pathway for PFASs, this study utilized a novel passive sampler, targeting a broad scope of PFASs—nonvolatile precursors, volatile neutral precursors, and legacy ionic PFASs—to enable a more comprehensive assessment of indoor exposure than most previous studies and demonstrated the sampler’s first use in a large-scale indoor campaign. This study also collected simultaneous measurements of PFASs across multiple matrices (water, air, and dust) alongside serum concentrations within the same population, with an increased sample size, number of compounds, and number of matrices compared to prior studies. The remote, isolated region enabled us to investigate background indoor exposure in the absence of significant local emissions. With impacted communities looking for ways to lower their exposure to PFASs, if indoor exposure was an important factor on the Faroe Islands, it could represent a potential opportunity for intervention, while identifying the importance of dietary pathways could guide mitigation in communities with highly contaminated diets.

## Methods

### Chemicals and Reagents

Liquid chromatography-grade methanol (LC-MeOH) and water (LC-water) were purchased from Fisher Scientific (Pittsburgh, PA, USA), along with ammonium hydroxide (NH_4_OH), ammonium acetate (C_2_H_7_NO_2_), ACS-grade ethanol (EtOH), ACS-grade methanol (MeOH), and ACS-grade ethyl acetate (EtOAc). Both native and mass-labeled analytical standards of all target analytes were purchased from Wellington Laboratories (Ontario, Canada) (see the SI for details).

### Passive Sampler Assembly

Polyethylene radial diffusive bodies, Radiellos, and Amberlite XAD-4 (Supelco) were purchased from Sigma-Aldrich. Sampling materials were precleaned with a rinse of basic MeOH and LC-MeOH before assembly. The Radiello bodies were filled with the XAD-4 sorbent and capped using a small amount of cotton wool and the Radiello vertical adapter. Prior to deployment, the samplers were stored in muffled (heated to 450 °C) aluminum foil packets and plastic bags for chemical and physical protection.

### Field Deployment

A subset of homes from cohort 5 (which recruited participants from subsequent childbirths during 2007–2009) was chosen through self-selection, with the first 40 responding homes selected. Air samplers were deployed in all 40 homes across the Faroe Islands in 2021 for approximately 28 days. Discrepancies in deployment time were due to logistical factors, but differences are accounted for in the calculations of concentrations. Samplers were placed in an elevated place (roughly eye level or above) in a well-ventilated space without direct sunshine, typically in the living room. Dust samples were collected by the households using a vacuum cleaner with a new vacuum bag inserted before the sampling period. Households were vacuumed prior to the sampling period and then regularly vacuumed during the 4-week sampling period. Ten 1 L drinking water samples using HDPE sampling bottles were also collected from 8 locations across the Faroe Islands (see the SI for details).

Questionnaires were conducted at 35 of the 40 homes (all 40 homes were asked, with 35 homes responding), collecting information on both the home (such as age, renovations, housing materials) and the occupants’ habits (such as consumer product use); see SI Table S16. Sample deployment and collection, as well as the collection of questionnaire data, was conducted under IRB Exempt Category 4­(iii) (IRB No. 00000599). All questionnaire participants provided informed consent prior to participation.

### Sample Extraction

Both air and dust samples were extracted based on previously published methods.^26,33,34^ Briefly, samples were spiked with mass-labeled internal standards and extracted using solid–liquid extraction (SLE). See the SI for full extraction method details. Water samples were extracted and analyzed using a modified EPA method 533 in accordance with methods detailed in the literature.^35,36^

### Instrumental Analysis

Air and dust samples were analyzed for neutral PFAS on an Agilent 7890B gas chromatograph coupled to an Agilent 5977A mass spectrometer (MS) detector operating in positive chemical ionization mode by using selected ion monitoring. Thermal transformation of nonvolatile PFAS to FTOHs during GC–MS analysis was not observed; see the SI for details. Dust and water samples were analyzed for ionic PFASs on a liquid chromatograph SCIEX ExionLC AC UHPLC coupled to a SCIEX X500R quadrupole time-of-flight tandem mass spectrometer (QTOF MSMS), following the previously published method.^35^ For additional details and quality assurance, refer to the SI.

### QAQC

The quantification of all targeted PFASs in samples and quality control (QC) samples was based on the isotope dilution method. The concentrations of the target compounds (except the polyfluoroalkyl phosphates (diPAPs), see the SI) were recovery-corrected by a set of mass-labeled internal standards spiked into each sample prior to extraction (see the SI, Tables S1 and S2). Recoveries of the surrogate mass-labeled PFAS spiked into the real samples, blanks, and quality control samples were within QC criteria of 60–140% (SI Table S7).

Instrumental quality control was performed by running a mid-calibration standard with every batch of analyzed samples. Process blanks and instrumental blanks were included in each sample batch for laboratory quality control. Travel blanks and field blanks were also included for air and water samples to monitor the sample preparation method performance. Water sample field blanks consist of exposing 250 mL of LC-water, taken from the lab prior to the sampling campaign, to the environment at sampling sites. Air passive sampler field blanks consist of exposing a precleaned passive sampler to the environmental conditions at sampling sites during the passive sampler’s deployment and collection. Method detection limits (MDLs) were calculated as the field blank average plus 3 times the standard deviation; however, when a compound was not detected in the blanks, 0.5 times the instrumental detection limits (IDLs) were used. MDLs were not calculated for target compounds that were not detected in any blanks or samples. See Tables S3 and S4 for MDLs. Only values above the MDLs were reported.

### Data Analysis

Volumetric air concentrations (ng/m^3^) were calculated from the quantified amount of a compound (ng/sample) by dividing by the deployment length (days) and sampling rate (m^3^/day). For the passive sampler, sampling rates for individual compounds have been previously determined and published for a select number of compounds. For XAD-passive samplers that do not utilize the polypropylene Radiello tubes, previous calibration studies have reported sampling rates between 0.1 m^3^/day and 0.6 m^3^/day for a wide range of semivolatile organic compounds (SVOCs), with only one PFAS included among evaluated compounds.^37^ For the Radiello-XAD samplers specifically, indoor calibration studies employing simultaneous active sampling were conducted.^26^ The samplers were also recently utilized as passive samplers in a large-scale outdoor atmospheric study, for which a single sampling rate of 0.1 m^3^/day was used.^38,39^ Based on these previously published deployments, and sampling rates calculated for the XAD-passive samplers, a sampling rate of 0.1 m^3^/day was used to derive air concentrations for all detected PFASs.^26,37,38,40^

Concentrations in all samples were blank-corrected using the average blank level (if detected in blanks). For statistical analysis, nondetects or values below the MDL in all sample matrices were replaced with $\mathrm{MDL}/\sqrt{2}$ . Spearman’s rank was used to assess the correlation between data sets. Only compounds with detection frequencies greater than 50% were considered as true and assessed for significance. All data visualizations and statistical analysis were done with R (R version 4.2.2).

The total estimated daily intake (EDI) for children aged 15 was calculated by using concentrations measured in this study. An age of 15 years old was chosen for EDI calculations to reflect the age at which blood samples were taken for cohort 5. Both indirect (from air and dust) and direct (from dust) exposures were considered, using biotransformation factors to calculate indirect exposure from precursors.^41^ For the purpose of comparison against EU guidelines on the food tolerable weekly intake, only indirect and direct exposure to 6 PFAAs (perfluorohexanoic acid (PFHxA), perfluorooctanoic acid (PFOA), perfluorononanoic acid (PFNA), PFDA, perfluorohexanesulfonic acid (PFHxS), and perfluorooctanesulfonic acid (PFOS)) were considered.

For the total EDI, eq 1 was used,

1 EDI=DirectPFAAintake+ΣIndirectPFAAintake

where direct perfluoroalkyl acids (PFAA) intake is calculated using eq 2,

2 $$\mathrm{Direct}\;\mathrm{PFAA}\;\mathrm{intake}=\Sigma \left(\frac{C_{\mathrm{PFAS}\mathrm{dust}}\times q_{\mathrm{dust}}\times f_{\mathrm{time}‐\mathrm{in}}\times F_{\mathrm{uptake}}}{\mathrm{BW}}\right)$$

where

C_PFASdust_ are the measured perfluorocarboxylic acids (PFCAs) and PFOS concentrations in dust samples (ng g^–1^);

*q*_dust_ is the quantity of ingested dust (g day^–1^);

*f*_time‑in_ is the fraction of time spent indoors;

*F*_uptake_ is the gastrointestinal uptake fraction; and

BW is the body weight (kg).

For eq 1, indirect PFAA intake is calculated using eq 3,

3 $$\mathrm{Indirect}\;\mathrm{PFAA}\;\mathrm{intake}=\Sigma \left(\frac{C_{\mathrm{neutral}\mathrm{PFAS}\mathrm{air}}\times q_{\mathrm{air}}\times f_{\mathrm{time}‐\mathrm{in}}\times F_{\mathrm{uptake}}\times F_{\mathrm{biotransformation}}}{\mathrm{BW}}\right)+\left(\frac{C_{\mathrm{neutral}\mathrm{PFAS}\mathrm{dust}}\times q_{\mathrm{dust}}\times f_{\mathrm{time}‐\mathrm{in}}\times F_{\mathrm{uptake}}\times F_{\mathrm{biotransformation}}}{\mathrm{BW}}\right)$$

where

C_neutralPFASair_ and C_neutralPFASdust_ are the concentrations of measured precursors in air (ng m^–3^) and dust (ng g^–1^);

*q*_air_ is the quantity of air inhaled (m^3^ day^–1^); and

*F*_biotransformation_ is the biotransformation constant of each precursor.

The EDI was calculated for three exposure scenarios: (i) “low”, representing the fifth percentile, (ii) “intermediate”, representing median concentrations, and (iii) “high”, representing the 95th percentile. For each exposure scenario, the corresponding percentile measured concentrations in air and dust were used. Inhalation and ingestion rates originated from the US EPA exposure handbook.^42,43^ For inhalation rates, the lower, median, and upper values given were used for each corresponding exposure scenario. In the case of dust ingestion, in which a lower rate was not given, the median value was used for both the low and intermediate scenarios. Uptake factors and biotransformation factors were compound- and scenario-specific and taken from previously published work.^41^ We assumed an average body weight of 56.8 kg^44^ and the total time spent indoors to be 90% (for more details, see the SI and SI Table S13).^6^

Blood serum concentrations for children were available for 33 homes. A one-compartment toxicokinetic (TK) model was used to assess the contribution of indoor exposure from air and dust to measured serum concentrations (for details, see the SI).^45,46^ The contribution of indoor exposure to blood serum concentrations for a specific PFAS compound was then calculated using eq 4:

4 $$\mathrm{Contribution}\;\left(\%\right)=\frac{\mathrm{Total}\;\mathrm{Predicted}\;\mathrm{Serum}\;\mathrm{Concentration}}{\mathrm{Actual}\;\mathrm{Serum}\;\mathrm{Concentration}}\times 100$$

## Results and Discussion

### PFAS Concentrations in Air

In the air samples, 6 neutral target PFASs were detected in at least one home, with fluorotelomer alcohols (FTOHs) dominating in both concentration level and detection frequency (d.f.) (Figure 1, Table 1, Table S8b). For example, 6:2 FTOH (d.f. 98%) ranged from 1.54 to 40.7 ng/m^3^ when detected, followed by 8:2 FTOH (d.f. 100%) ranging from 0.79 to 15.1 ng/m^3^, and 10:2 FTOH (d.f. = 98%) ranging from 0.26 to 4.19 ng/m^3^ when detected (Table 1, Table S8b). FTOH concentrations were not influenced by the presence of diPAPs during the GC–MS analysis, as confirmed through the thermal transformation test (see the SI). *N*-Ethyl perfluorooctanesulfonamido ethanol (EtFOSE) was detected in approximately 50% of the homes but at lower concentrations (0.06 to 0.09 ng/m^3^) as compared to the FTOHs (Table 1, Figure 1). Also detected in the air samples at relatively low concentrations were fluorotelomer acrylates, 8:2 FTAcr (0.49 to 0.58 ng/m^3^) and 10:2 FTAcr (0.38 to 0.44 ng/m^3^), with detection frequencies of approximately 10% (Table 1). For more details, see the SI. Previous studies have reported significant relationships between consumer products and volatile PFAS concentrations in indoor air, indicating that possible sources inside these homes may include products such as outdoor clothing or furniture.^47,48^

**Figure 1:**
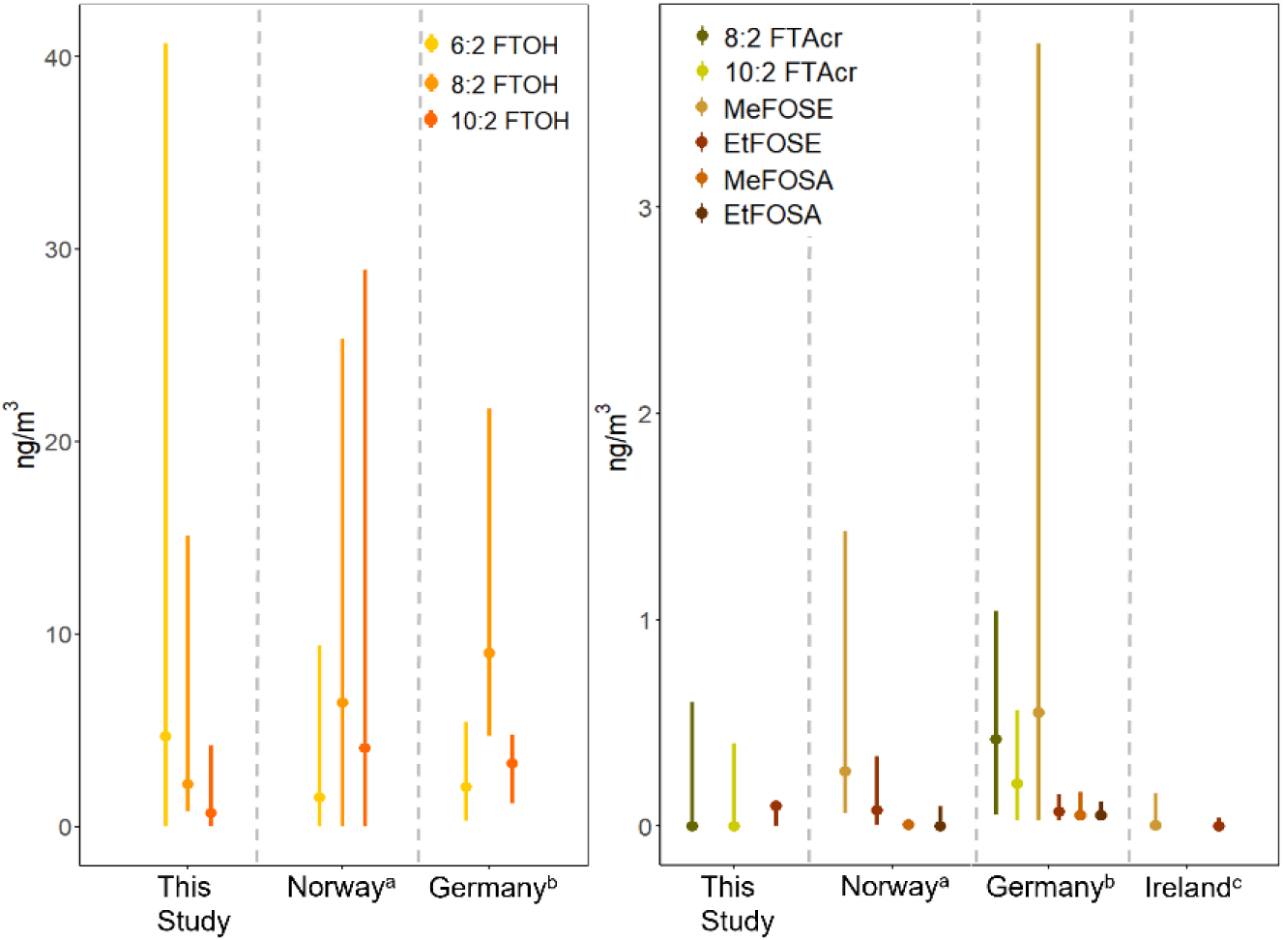
Concentrations of neutral PFASs measured in the air of homes from this study in comparison to other European countries. Points represent median values, with lines representing the range. ^a^Haug et al., 2011. ^b^Fromme et al., 2015. ^c^Harrad et al., 2019.

**Table 1:** Summary of Neutral PFAS Air Concentrations^a^

Compound	Minimum (ng/m^3^)	Median (ng/m^3^)	Maximum (ng/m^3^)	Detection frequency (%)
6:2 FTOH	<1.54	4.75	40.7	98
8:2 FTOH	0.785	2.18	15.1	100
10:2 FTOH	<0.259	0.728	4.19	98
8:2 FTAcr	<0.491	0.491	0.575	10
10:2 FTAcr	<0.381	0.381	0.436	13
MeFOSA	ND	ND	ND	0
EtFOSA	ND	ND	ND	0
MeFOSE	ND	ND	ND	0
EtFOSE	<0.061	0.062	0.085	53

a ND: nondetects.

Previous studies conducted across Europe have reported similar concentrations of PFASs in indoor air (Figure 1). For example, FTOHs in air were reported for Norwegian and German homes at the same magnitude as we measured across Faroe Island homes (Figure 1, Table S10).^11,12^ In both studies, 8:2 FTOH had the highest median concentrations, followed by 10:2 FTOH, with 6:2 FTOH having the lowest median concentration of the FTOHs (Figure 1).^11,12^ The change from 8:2 FTOH being the most dominant in the earlier studies to 6:2 FTOH in this study may be a result of product shift over time.

Also detected in the Norwegian and German homes were perfluorinated sulfonamides, reporting similar concentrations of EtFOSE to those found in this study (Figure 1). However, both studies also reported detection of other perfluorinated sulfonamides (MeFOSE, MeFOSA, and EtFOSA), which this study did not detect in the air (Figure 1). Another study conducted in Irish homes also reported EtFOSE at similar concentrations to those found in the Faroes but again found the presence of MeFOSE also (Figure 1).^49^ This shift in detection of FOSEs and FOSAs likely reflects the phasing out of PFOS-related chemicals since 2002,^1^ and so, lower detection is expected.

### PFAS Concentrations in Dust

In dust samples, 8 neutral target PFASs were detected in at least one home with 6:2 FTOH having the highest median concentration (26.6 ng/g dust) with a range of 17.6 to 94.3 ng/g dust (Table 2, SI Table S9d). MeFOSA (12.3 to 16.7 ng/g dust) and EtFOSA (10.4 to 17.8 ng/g dust) were the most frequently detected, followed by MeFOSE (15.3 to 23.2 ng/g dust) and the FTOHs (∑FTOHs 14.5 to 180.1 ng/g dust) (Table 2, SI Table S9d). 10:2 FTAcr was also detected in around 60% of the homes (9.7 to 14.5 ng/g dust) (Table 2, SI Table S9d). For more details, see the SI. Possible sources of these volatile PFASs in the dust include textiles and fabrics with stain-resistant or water-repellent treatments, cleaning products, and other consumer products.^48,50,51^

**Table 2:** Summary of Neutral and Ionic PFAS Dust Concentrations^a^ ^b^

Compound	Minimum (ng/g dust)	Median (ng/g dust)	Maximum (ng/g dust)	Detection frequency (%)
6:2 FTOH	<0.176	26.6	94.3	88
8:2 FTOH	<0.176	16.3	93.3	73
10:2 FTOH	<0.176	0.176	49.2	33
8:2 FTAcr	ND	ND	ND	0
10:2 FTAcr	<0.176	10.5	14.5	63
MeFOSA	<0.176	14.2	16.7	98
EtFOSA	<0.176	14.0	17.8	98
MeFOSE	<0.176	17.0	23.2	83
EtFOSE	<0.176	0.176	19.5	35
PFHxA	0.522	2.19	14.3	100
PFOA	0.493	1.61	16.7	100
PFNA	0.145	0.359	2.61	100
PFDA	0.207	0.540	9.25	100
PFHxS	<0.004	0.004	1.51	15
PFOS^b^	<0.023	1.98	24.2	85
6:2 diPAP	<0.071	4.80	48.8	98
8:2 diPAP	<0.007	0.47	8.10	70

a Only ionic compounds used for the EDI calculations are shown. ND: nondetects.

b Branched and linear sum

Other studies in Europe displayed a wide range of results. For example, the median sum of FTOHs in this study was approximately 2 and 100 times greater than that reported for Germany and France, respectively (Figure 2, right).^14,52^ For the sum of FOSEs/FOSAs, this study’s median was 100 times smaller than reported for Spain^17^ but 100 times greater than that reported for France (Figure 2, right).^52^ The Faroe Islands’ precursor concentrations in dust were the closest to those reported in Finland (Figure 2, right).^18^

**Figure 2:**
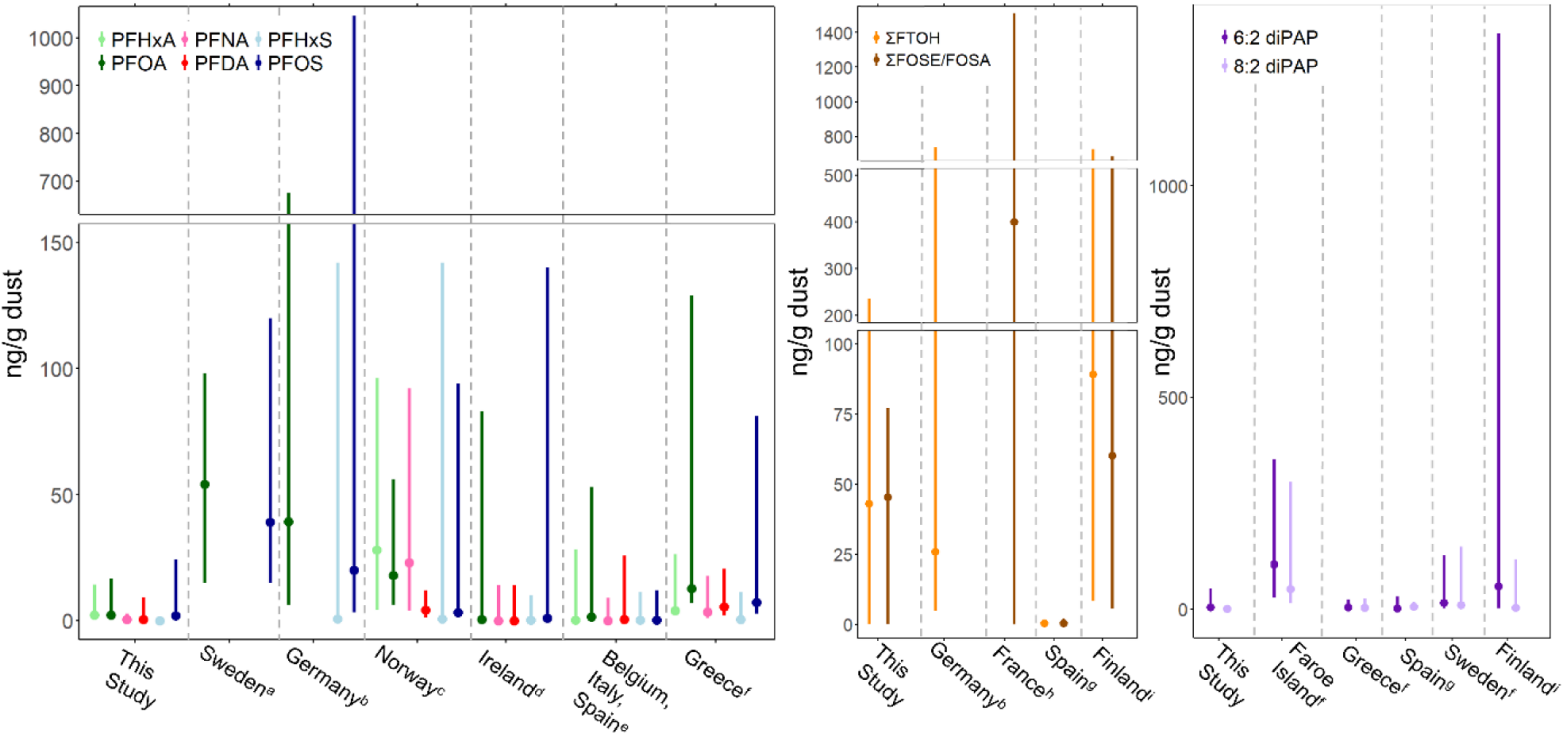
Concentrations of ionic (left) and neutral (middle) PFASs and diPAPs (right) measured in the dust of homes from this study in comparison to other European countries. Points represent median values, with lines representing the range. Only ionic compounds are factored into the estimated daily exposure shown. ∑FTOH = Sum of 6:2 FTOH, 8:2 FTOH, and 10:2 FTOH. ∑FOSE/FOSA = Sum of MeFOSA, EtFOSA, MeFOSE, and EtFOSE. ^a^Bjoerklund et al., 2009. ^b^Xu et al., 2013. ^c^Haug et al., 2011. ^d^Harrad et al., 2019. ^e^De La Torre et al., 2019. ^f^Eriksson and Kärrman, 2015. ^g^Ericson Jogsten et al., 2012. ^h^Goosey and Harrad, 2011. ^i^Winkens et al., 2018.

Ionic PFASs were only measured in dust samples, with 20 target compounds detected in at least one home (see SI Table S9b for the full list of detected compounds). PFCAs (C4–C14) were the most frequently detected, found in 100% of the homes (Table 2, SI Table S9d). PFOS and perfluorodecanesulfonic acid (PFDS) were also commonly detected, found in at least 85% of homes (Table 2, SI Table S9d). Of the PFAAs, PFDS was detected at the highest concentration (0.95 to 12.2 ng/g dust), followed by PFHxA (0.52 to 14.3 ng/g dust) and PFOS (0.31 to 24.2 ng/g dust) (Table 2, Table S9d). DiPAPs were also frequently detected in dust samples, with 6:2 diPAP (0.28 to 48.8 ng/g dust) detected in 98% of homes and 8:2 diPAP (0.12 to 8.1 ng/g dust) in 70% (Table 2, SI Table S9d). Among all target ionic PFASs, 6:2 diPAP was observed at the highest concentrations (Table 2, SI Table S9d). For full details, see the SI. PFAS concentrations measured in dust from these homes may indicate contributions from various indoor sources, particularly consumer products and treated textiles or materials.^8,53^

The median concentrations of PFOA and PFOS measured in dust samples across the Faroe Islands are 5 to 35 times smaller than those measured by previous studies at homes in other European countries (Figure 2, left).^13,14^ The measured median concentrations and ranges of other PFAAs (PFHxA; perfluorononanoic acid, PFNA; perfluorodecanoic acid, PFDA; and perfluorohexanesulfonic acid, PFHxS) in the dust of Faroese homes were also generally smaller than those reported for other European homes (Figure 2, left).^11,15,16,49^

A previous study by Eriksson and Kärrman, 2015, measured a range of polyfluoroalkyl phosphate esters (PAPs) in dust from a small number of homes (*n* ≤ 10) in several countries, including the Faroe Islands.^16^ For dust sampled from the Faroe Islands, compared to concentrations found in this study, they reported median concentrations that were 20 times higher for 6:2 diPAP and 100 times higher for 8:2 diPAP (Figure 2, Table S10).^16^ The previous study also collected dust samples from other European countries (Greece, Sweden, and Spain), with their reported concentrations being similar to those found in samples collected in this study (Figure 2, Table S10).^16^

### PFAS Concentrations in Water

All target compounds were below the limit of detection in any collected water sample (see the SI, Table S4). Low detections of PFAS in water were expected, given the absence of manufacturing of PFAS or production of consumer products throughout the Faroe Islands, the remote location, and the low concentrations in water samples reported previously.^54^

### Air–Dust Correlations

Correlations between concentrations measured both within the same matrices (air–air and dust–dust) and across the different matrices (air–dust) were investigated. Strong positive correlations (*R* ^2^ > 0.7) were found between 12 pairs of compounds (*p* < 0.01, Table S11). The strongest correlations were found between 8:2 FTOH and 10:2 FTOH in the air and PFOA and PFDA in the dust (*R* ^2^ = 0.93, Table S11). Other strong positive correlations were found among C5–C10 PFCAs and between MeFOSA and EtFOSA in the dust (Figure 3). These correlations suggest that common sources of these compounds were present in homes; 8:2 FTOH and 10:2 FTOH (and MeFOSA and EtFOSA) were possibly from the same legacy products.^55^ As both long-chain FTOHs and FOSAs have been phased out, the detection of these compounds likely represents legacy products sold before 2002.^1^ Prior work, such as for example, Haug et al., 2011, observed strong, significant correlations among FTOHs in the indoor air of Norwegian homes.^11^ Strong correlations between PFCAs in dust have been observed in numerous studies globally, such as the Czech Republic, Canada, and the USA.^34,56^

**Figure 3:**
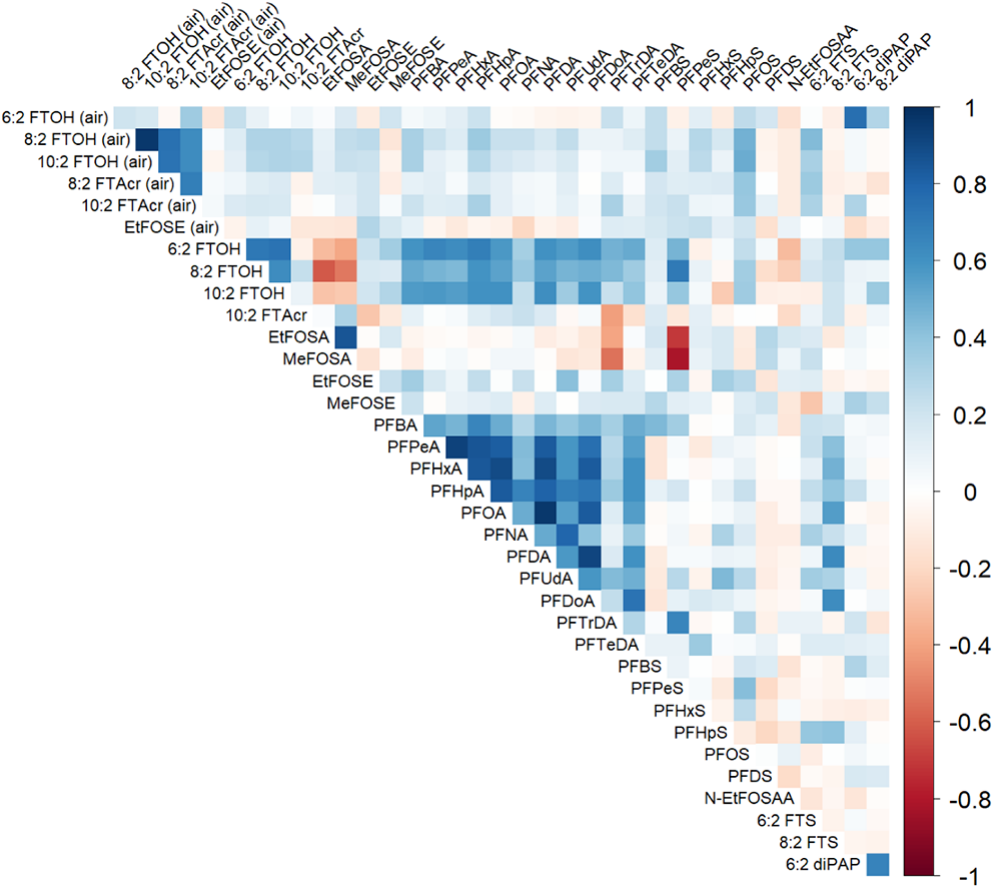
Correlation heat map with all compounds detected in both air and dust. *­(air): Neutral compounds measured in air; all other compounds measured in dust. For corresponding *R* ^2^ values, see SI Table S11.

A strong, significant correlation (*R* ^2^ = 0.76, *p* < 0.01) was observed between 6:2 diPAP measured in dust and 6:2 FTOH measured in the air (SI Table S11), which may indicate the formation of the FTOH from the precursor. The transformation pathway of diPAPs yields FTOHs, which then further degrade to the PFCAs.^16^ Weaker correlations were observed between 8:2 diPAP in the dust and 6:2 FTOH in the air (*R* ^2^ = 0.30) and also between 6:2 diPAP and 8:2 diPAP in the dust (*R* ^2^ = 0.66). The same relationships were noted by Winkens et al., 2018, in which samples collected from Finnish homes were analyzed for diPAPs among PFAAs and other precursor PFASs.^18^ As discussed by Winkens et al., 2018, these correlations, particularly the correlation seen between 6:2 diPAP and 8:2 diPAP, are likely indications of common sources in the home.^18^

Weaker correlations (0.4 < *R* ^2^ < 0.7) were observed between other precursors measured in the air, for example, between 8:2 FTOH and 10:2 FTOH (SI, Table S11). Similar correlations were also seen between the same precursor compounds in dust (SI Table S11). Correlations between precursors indicate the use or presence of consumer products and goods that contain mixtures of similar compounds.^55^ In dust, weak correlations (0.1 < *R* ^2^ < 0.5) existed between the precursors and PFAAs and 8:2 fluorotelomer sulfonic acid (8:2 FTS) and some PFAAs (such as PFOA, PFDA, and perfluorododecanoic acid (PFDoA)) (SI, Table S11). While correlations are weak, they could be an indication of the use of 8:2 FTSs as a replacement for the PFAAs. The correlations between the neutral precursors and PFAAs may be a result of their potential transformations.^57–61^ This evidence of precursor transformation that we observe in the dust emphasizes the importance of including indirect exposure from precursors in total exposure calculations. While correlations between precursors in the air and PFCAs in the dust would be further evidence of transformation, much like this study, Haug et al., 2011, also reported no such correlations.^11^ The lack of significant correlations in this instance may indicate that the PFCAs in homes originate from both direct sources in addition to transformation of precursors. For more details on other noted correlations, see the SI.

### Air–Dust Partitioning

Dust–air partition coefficients (log *K*_dust–air_) were calculated for the 5 precursor compounds detected in both air and dust samples and correlated with octanol–air partition coefficients (log *K*_oa_) from Dreyer et al., 2009, and Wang et al., 2011 (SI, Table S12).^62,63^ A log *K*_dust–air_ log *K*_oa_ relationship was observed (*y* = 0.45*x* – 1.43, *R* ^2^ = 0.916, *p* < 0.01) (Figure S2, shown in black). This suggests that the partitioning between gas-phase compounds in the air and their sorption to the organic dust control their distribution between the two matrices in homes. These results also imply that the sampling protocol employed in this study did not significantly introduce bias into the results.

In previous studies, similar significant relationships between log *K*_dust–air_ and log *K*_oa_ were found for both precursors and PFAAs (Figure S2, shown in red).^18^ Winkens et al., 2018, used a similar sized sample set of 55 homes in Finland to look at the relationship for four precursor compounds and six PFAAs.^18^ While their reported slope of 0.55 is already quite similar to the relationship found in this study of 0.45 (±0.080), upon removal of PFAAs from their plot, the relationship for just their reported precursor compounds changes to *y* = 0.47*x* – 1.31, *R* ^2^ = 0.925 (Figure S2, shown in blue). This indicates that the relationship between dust–air partitioning and log *K*_oa_ may be better represented with separate linear regression lines for precursors (Figure S2, shown in blue) versus ionic PFASs (Figure S2, shown in green). Such relationships indicate that it may be possible to estimate concentrations in air from measured dust samples (or vice versa), as also reported by previous studies.^18,64–66^ However, confidence in such predictions is currently limited by data availability and the small number of PFASs detected in both phases concurrently.

### Questionnaire Data

Questionnaires collected data on both homes, such as age of house and materials within home, as well as occupants’ habits, such as consumption of take-out food and use of PFAS-containing products (see the SI, Table S16). Overall, significant variability between homes could not be identified, and so, no strong correlations emerged between questionnaire answers and PFAS concentrations measured. Home characteristics and personal habits were generally similar across all 40 homes. For example, only 2 homes had carpets and almost all homes contained upholstered furniture. This data was unsurprising, given that many residences share similar styles and construction materials, likely reflecting both limited local resources and the high cost of imported building supplies. The room in which the air sampler was placed varied between the kitchen and living room, but regardless, in most cases, the room had windows that were often opened. Some participants were able to confirm the presence of PFAS-containing products in their home; however, many did not know what PFASs are or which products contain PFASs (SI, Table S16).

### Estimated Daily Intake

Total EDI (from indirect and direct exposure to PFHxA, PFOA, PFNA, PFDA, PFHxS, PFOS) was 0.001 ng/kg bw for low exposure, 0.012 ng/kg bw for intermediate exposure, and 0.129 ng/kg bw for high exposure (Figure 4). For the low exposure scenario, direct and indirect exposures from dust contribute similarly and together account for 97% of the total exposure (Figure 4). Unlike the low exposure scenario, for intermediate and high exposure scenarios, indirect exposure from air contributes about 65% to the total exposure (Figure 4). For the intermediate and high exposure scenarios, where indirect exposure to volatile, neutral precursors contributed more than direct exposure to PFAAs, the detected FOSEs and FOSAs had the largest contribution in dust, while in air, the FTOHs played a larger role. Exposure to diPAPs contributed significantly to total exposure only in the high exposure scenario. For direct exposure to PFAAs, PFHxA, PFOA, and PFOS were the most significant compounds. Our results imply that volatile, neutral PFAS are major contributors to total exposure in children from dust ingestion and air inhalation pathways, in line with other studies investigating children’s exposure to PFASs in indoor environments.^26,67^

**Figure 4:**
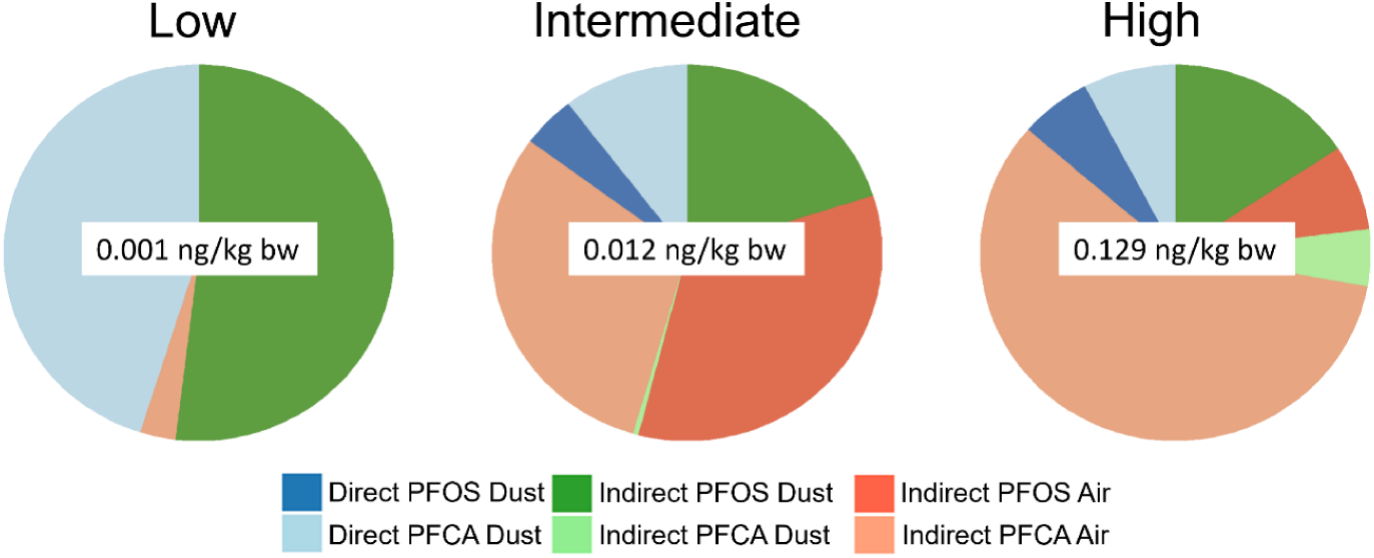
EDI for children in low, intermediate, and high exposure scenarios, showing contributions to PFOS and ΣPFCA from different exposure pathways.

The EDIs calculated in this study for the Faroe Islands are slightly lower than those calculated for other European studies. For example, the EDI calculated from dust and air concentrations of PFAAs (PFHxA, PFOA, PFNA, PFDoA, perfluoroundecanoic acid (PFUnDA), PFOS, and 8:2 fluorotelomer sulfonic acid (8:2 FTSA)) and their corresponding precursors measured in Finland were 0.027 and 0.019 ng/kg bw (for a total of 0.046 ng/kg bw) for the intermediate scenario.^18^ A study by Gustafsson et al., 2022, calculated EDI from the ingestion and inhalation of dust in homes in Stockholm, Sweden. For the sum of the four PFASs (PFOA, PFNA, PFHxS, and PFOS), they reported an EDI of 0.240 ng/kg bw and 0.483 ng/kg bw for median and high exposure scenarios, respectively.^68^

### Contribution to Total Exposure

To estimate the importance of air inhalation and dust ingestion relative to other pathways, we can use the current EU guidelines for a PFAS tolerable weekly intake (TWI), set by the European Food Safety Authority (EFSA) in September 2020, of 4.4 ng/kg bw/week for four PFASs (PFOA, PFNA, PFHxS, PFOS).^69^ On this basis in an intermediate scenario for children, EDI from dust ingestion contributes 1% to the TWI, and air inhalation 1% (Figure S3). For a high exposure scenario, contributions from dust and air are around 10 times higher, at 7% and 13%, respectively (Figure S3).

While many families in the Faroe Islands still rely on a traditional diet, which has been reported to contain high concentrations of PFAS, consumption of pilot whale by children has declined over the past couple of decades.^28^ For those who do include pilot whale regularly in their diet, the average consumption of pilot whale for a child was approximately 0.08 g/kg bw/day.^28^ Based on reported concentrations measured in pilot whale by Dassuncao et al., 2017, a child’s average consumption would equate to an EDI of 0.38 ng/kg bw or 60% of the TWI (Figure S3).^70^

These results suggest that for a child at any indoor exposure level with a diet that incorporates pilot whale, dietary sources of PFAS may be more influential for total exposure, followed by dust ingestion and then air inhalation (Figure S3). In addition, there are additional exposure pathways, linked to consumer products, such as cosmetics,^71^ and the presence of PFASs in other food items beyond whale meat. While estimations of exposure from other dietary sources were beyond the scope of this study, previous studies have calculated the EDI from typical diets in other European countries. The dietary intake for the four PFASs considered in the EU TWI (PFHxS, PFOS, PFOA, and PFNA) for 11 reported countries ranged from 0.5 to 60 ng/kg bw/day.^72^ While some variation may be explained by methods used and which (or how many) matrices were examined, the EDI from an average diet varies greatly depending on the country, and some countries’ estimates even exceed the EU TWI. Furthermore, while the Faroe Islands were not one of the countries reported, it is still evident that a large focus should be placed on diet for communities wanting to reduce their total exposure to PFASs.

### Predicted Serum Concentrations

Serum concentrations were reported for 9 PFAAs in children from 33 of the 40 homes sampled for PFASs in air and dust. Of the compounds measured in serum, 6 (PFHxS, PFOA, PFNA, PFDA, PFUnDA, and PFOS) were frequently detected (d.f. ≥ 85%). Indoor PFAS concentrations measured in this study were used to predict serum concentrations, using a one-compartment TK model as described in the methods. Predicted serum concentrations were compared to actual serum concentrations to assess the contribution of indoor exposure. Across the 33 households, indoor exposure accounted for less than 5% of the actual measured serum concentration in the low exposure scenario (Figure 5). For the high exposure scenario, indoor exposure accounted for 8% of the actual serum PFOS concentration and around 20% for both PFOA and PFDA (Figure 5). While we could only account for direct exposure from dust for PFNA, we observed that direct exposure from dust and indirect exposure from air played a large role for PFOA and PFDA. However, for PFOS, indirect exposure from dust played a larger role across all three scenarios (Figure 5). While these results provide insight into which indoor exposure pathways may contribute to blood serum concentrations of PFAS, they also highlight the importance of other pathways, such as diet, that may play a larger role in total exposure.

**Figure 5:**
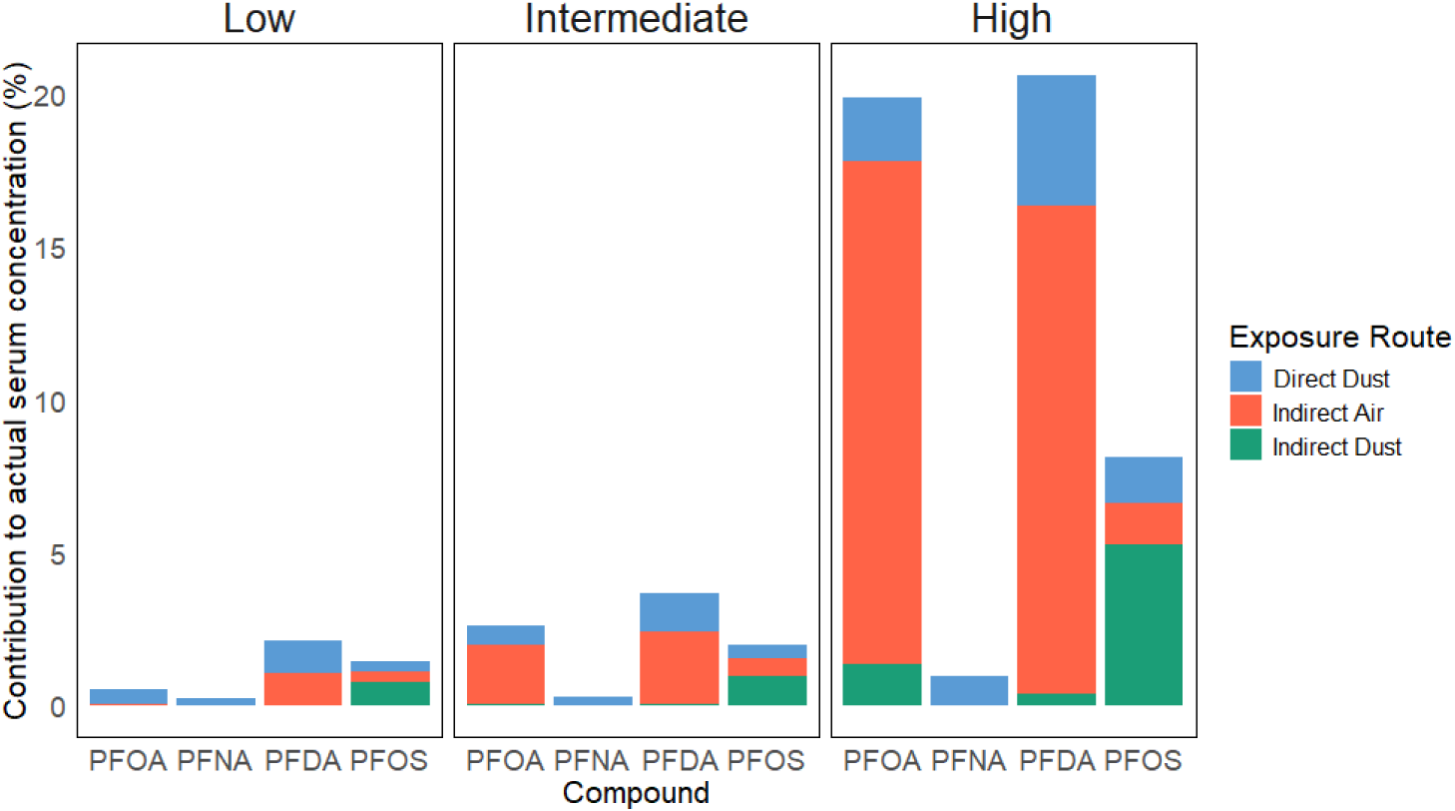
Average contribution (%) of indoor exposure to actual serum concentrations measured for 4 compounds, PFOA, PFNA, PFDA, and PFOS, for the three exposure scenarios.

### Limitations and Implications

There are several limitations of this study. First, the use of the novel passive sampler introduces uncertainty into the atmospheric concentration calculations. Although this study relied on previously reported sampling rates, compound-specific sampling rates were not available. Future applications should include further calibration and validation to establish compound-specific sampling rates to reduce this uncertainty. Second, the assumptions made and uncertainties in variables used for the EDI calculation must be considered when interpreting the EDI results. For example, the time spent inside the home was assumed to be the same across all households, and while this general assumption is often made in EDI assessments, it does not allow us to tailor the EDI to the specific household. Another challenge with EDI calculations is the uncertainty in variables such as biotransformation factors. While this study employed the best practice for the exposure assessment, higher confidence in these variables could lead to more accurate exposure assessments in the future. Finally, the authors did not collect household-specific dietary data as it was considered outside the scope of this study. An estimate of the most recent available data was used for consumption of pilot whale; however, the data is somewhat outdated and is not household-specific.

Despite these limitations, the findings from this study do allow us to highlight both areas of progress and areas that should be the focus of future research. First, this is the first published use of these passive air samplers in a campaign designed to simultaneously measure indoor air concentrations across a large number of homes, demonstrating their ease of use in studies such as this. Second, the relationship between *K*_dust/air_ and *K*_oa_ for the detected precursors in our study presents the possibility to both estimate *K*_dust/air_ of other precursors not detected in this study based on their *K*_oa_ and predict the concentration of the compound in dust if only air is measured (or vice versa). This finding supports and expands upon the conclusions from previous studies, such as Winkens et al. (2018).^18^ However, further investigation of dust–air partition coefficients for PFASs is needed, given the limited available data and relatively small range of PFASs simultaneously detected in both phases. The correlations between compounds in air and dust matrices, along with comparison against older studies conducted in homes, are evidence of the changing compounds in use, reflecting replacement compounds as a result of regulations and bans. Finally, in the larger picture of total exposure to PFASs, consuming pilot whale remained the most significant factor, despite average consumption declining over the past few decades. This emphasizes the importance of reducing pilot whale consumption and highlights dietary exposure as an area of focus for remote communities, such as the Faroe Islands, that want to reduce their exposure to PFASs. However, this observation is only applicable to those who include pilot whale in their diet. In cases where pilot whales are not consumed at all, and if average consumption continues to decline, exposure to PFASs from indoor matrices will be more significant and could be a future focus for reducing total exposure.

## Supplementary Material





## Notes

Tables on GC–MS target compounds, LC–MS target compounds, GC–MS MDLs, LC–MS MDLs, PFAS recovery from SRM dust, diPAP recovery test, matrix recovery, air concentrations, dust concentrations, diPAP concentrations, air–dust *R*-squared values, air–dust partitioning, EDI parameters, EDI from indirect exposure to precursors via air and dust, total EDI, questionnaire data, serum concentration calculation parameters, and serum concentration contribution (XLSX)
Additional details on analytical methods, standards, quality control and assurance, instrument conditions, individual household air and dust concentrations, statistical analysis, intercountry comparison, EDI calculations, and questionnaire data (PDF)
