# Supplementary material for: Contribution of Indoor Air and Dust to Children’s Per- and Polyfluoroalkyl Substance Exposure in the Faroe Islands
Source: Environ Sci Technol. 2026 Jul 30;60(31):21804–14. doi: 10.1021/acs.est.6c02003 (PMC13472078; doi:10.1021/acs.est.6c02003)
Supplement: Supplementary file 1 [file es6c02003_si_002.pdf]

Supplementary Information

Contribution of Indoor Air and Dust to Children's Per- and Polyfluoroalkyl Substances Exposure in the Faroe Islands

Melissa Woodward[1], Jitka Becanova[1], Simon Vojta[1], Pál Weihe[2], Philippe Grandjean[3,4], and Rainer Lohmann[1,*]

[1]University of Rhode Island Graduate School of Oceanography, Narragansett, RI 02882.

*rlohmann@uri.edu

[2]Department of Occupational Medicine and Public Health, Sigmundargøta 5, 100, Tórshavn, Faroe Islands.

[3]National Institute of Public Health, University of Southern Denmark, Copenhagen, Denmark.

[4]Department of Biomedical and Pharmaceutical Sciences, University of Rhode Island, Kingston, RI 02881.

The supplementary information includes an excel data file with 22 tables, and a word document with 18 pages and 4 figures.

Table S1. Full target compound list in GCMS analysis method with associated mass-labeled internal standards.

Table S2. Full target compound list in LC/MS-MS analysis method with associated mass-labeled internal standards.

Table S3. List of neutral PFAS detection limits.

Table S4. List of ionic PFAS detection limits.

Table S5. Recoveries of known PFAS from NIST SRM 2585 Dust

Table S6. Extraction recoveries of diPAPs in dust matrix spike test.

Table S7. Recoveries of mass-labeled internal standards in samples.

Table S8a. Measured neutral PFAS concentrations in air samples.

Table S8b. Measured neutral PFAS concentrations in air summary: ranges, medians, and detection frequencies.

Table S9a. Measured neutral PFAS concentrations in dust samples.

Table S9b. Measured ionic PFAS concentrations in dust samples.

Table S9c. 6:2 diPAP and 8:2 diPAP concentrations and internal standard recoveries in dust samples.

Table S9d. Measured neutral and ionic PFAS concentrations in dust summary: ranges, medians, and detection frequencies.

Table S10. Air/Dust concentrations from other European studies.

Table S11. Air-Dust sample R-squared values and significance.

Table S12. Air-dust and octanol-air partitioning coefficients.

Table S13. Parameters used for EDI calculations from indirect exposure via air and dust.

Table S14. EDI from indirect exposure to precursors via air and dust.

Table S15. Total EDI from direct and indirect exposure via air and dust.

Table S16. Questionnaire Data.

Table S17. Parameters used for calculating serum concentrations from dust and air concentrations.

Table S18. Contribution (%) to serum concentrations from indoor air and dust concentrations summary (averages, SD).

Water Sampling

Ten 1 L drinking water samples were collected from 8 locations around the Faroe Islands (Figure S1), with 2 samples each collected from Hoyvík and Tórshavn. Samples from Havnardalur and Villingadalur were taken from drinking water supply reservoirs. All other samples were taken from tap water.

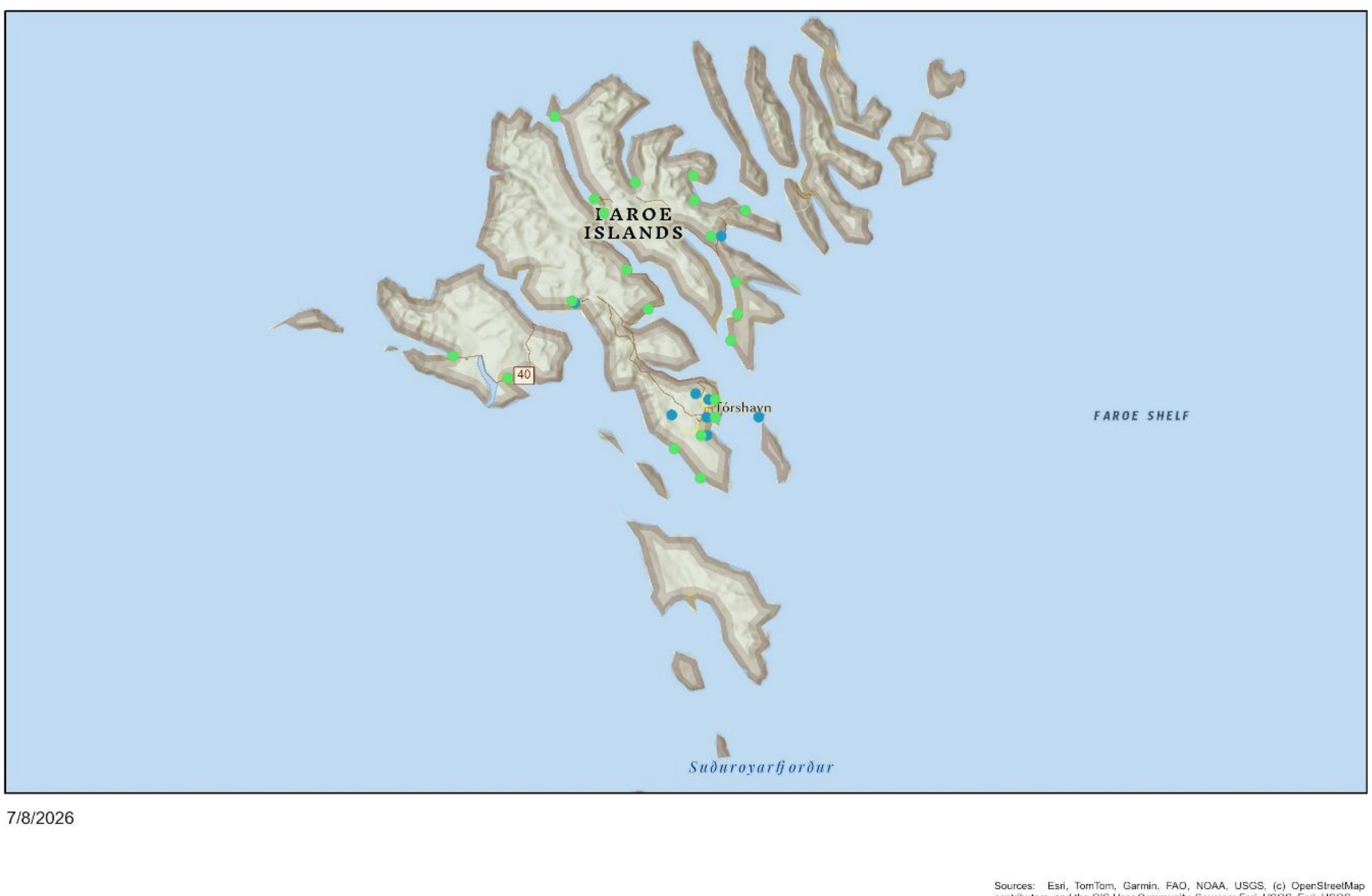

Figure S1. Sampling locations for collected water samples (blue) and air/dust sampling (green). Map created using ArcGIS.

Dust Sample Extraction

Two subsequent fractions, an LC-fraction and GC-fraction, were taken from each dust extract based on previously published methods.[1,2] Extraction efficiency of the method was tested using NIST SRM 2585 (SI Table S5). All extraction materials were pre-cleaned with a basic MeOH and LC-MeOH rinse. Dust samples were sieved with a 150 µm pore size to remove dirt and hair. 100 mg of dust was spiked with mass-labelled internal standards (SI Table S1 and Table S2) in a 15 mL pre-cleaned Falcon tube and solid-liquid extraction (SLE) was performed with 4 mL LC-grade MeOH. Samples were sonicated for 30 minutes and centrifuged at 4000 rpm for 5 minutes. The supernatant was removed and added to a pre-labelled, pre-cleaned Falcon tube. The extraction was repeated twice more for a total of 12 mL of MeOH extract. The sample was frozen overnight to separate any lipid present and cleaned via 0.5 g Supelclean™ ENVI-Carb™ cartridges. The extract was concentrated to approximately 0.5 mL in volume under a gentle stream of nitrogen. For LC analysis, an aliquot of this final concentrate was diluted with 4 mM ammonium acetate in water to achieve 40/60 of methanol/water ratio, suitable for analysis via liquid chromatography tandem mass spectrometry (LC-MS/MS). For GC analysis, the sample was further evaporated to 100 µL in volume and prepared for analysis via gas chromatography mass spectrometry (GC-MS).

Air Sample Extraction

Samples were extracted using SLE based on the method previously published.[3] The sorbent was removed from the diffusive tube and spiked with mass-labelled internal standards

(SI Table S1) in a 15 mL Falcon tube and SLE was performed using 4 mL of EtOAc. Samples were vortexed and sonicated for 30 minutes. After 24 h, the extract was collected in a pre-labelled, pre-cleaned Falcon tube. The SLE was repeated twice more, resulting in 12 mL total of extract. The extract was concentrated to 100 µL in volume and prepared for analysis via gas chromatography mass spectrometry (GC-MS).

Analysis of volatile and neutral PFAS

Prior to analysis, all samples were spiked with a recovery standard. Samples were analyzed using a gas chromatograph (GC) Agilent 7890B coupled with a mass spectrometer (MS) Agilent 5977A operating in positive chemical ionization mode using selected ion monitoring. The ion source was held at 300 °C, the transfer line was held at 280 °C, and the spitless intake was held at 270 °C. Sample aliquots of 1 µL were injected via an autosampler. Target compounds were separated on a polar 60 m Supelcowax 10 column. Gas flow of the helium carrier gas was held at 1.5 mL min^{-1}. The oven temperature was as follows: 50 °C for 2 min, 3 °C per min to 70 °C, 10 °C per min to 130 °C, 20 °C per min to 220 °C, 120 °C per min to 270 °C, hold for 5 min.

Previous studies have observed thermal transformation of nonvolatile PFAS to FTOHs in the high inlet temperatures during GC-MS analysis, resulting in falsely elevated concentrations of FTOHs.[4,5] To investigate, standards of 6:2 perfluoroalkyl phosphate diester (6:2 diPAP) and 8:2 perfluoroalkyl phosphate diester (8:2 diPAP) were injected into the GC-MS following the same method as for the volatile target analytes. Standards of up to 500 pg/µL were ran, but no thermal observation to FTOH was observed.

Analysis of ionic PFAS

Prior to analysis, all samples were spiked with recovery standards. The instrumental analysis was performed using a liquid chromatograph (LC) SCIEX ExionLC AC UHPLC coupled with a quadrupole time-of-flight mass spectrometer (QTOF MSMS) SCIEX X500R. A Phenomenex Gemini 3 µm C18 110Å 50 x 2 mm LC analytical column preceded with a Phenomenex SecurityGuard cartridge was used for the analyte separation. Another Phenomenex Gemini 5 µm C18 110 Å 50 x 4.6 mm LC analytical column was used to delay the PFAS instrumental contribution. The aqueous mobile phase (MPA) was 10 mM ammonium acetate in water, and the organic mobile phase (MPB) was 10 mM ammonium acetate in methanol. LC parameters were set to: flow 0.3 mL min^{-1}, injection 20 µL, column oven 45 °C. Solvent gradient of MPB gradually increased from 40 % to 80 % (1 to 5.5 min), 80 % to 100 % (5.5 to 7 min), then hold for one minute and finally drop to 40 % (8 to 8.5 min) and hold for another 6.5 minutes. For the quantification of the target analytes, a HRMSMS (MRM HR) method was used. Negative mode ESI with the following parameters was used: curtain Gas at 30 psi, ion source gas 1 at 40 psi, ion source gas 2 at 60 psi, temperature 450 °C.

Analysis of diPAPs

The inclusion of diPAPs as target compounds happened after the original extraction and analysis of the dust samples. Extraction efficiencies of diPAPs were hence determined through a matrix spiked extraction test, in which dust samples were spiked with a known amount of diPAPs

and recovery was 92 % (±12) and 78 % (±4) for 6:2 diPAP and 8:2 diPAP (SI Table S6). To quantify diPAPs in the actual dust samples, diPAP mass-labeled compounds were used as a non-extracted internal standard. Recoveries were calculated using an average of three other internal standard compounds that behaved simarly to the diPAP mass-labeled compounds in the matrix spike test, a similar approach to previous studies.[6] Recoveries for each sample are reported in the SI (SI Table S9c), but not used to recovery correct concentrations.

Estimated Daily Intake (EDI) Calculations

For EDI calculations, low (5th percentile), intermediate (median), and high (95th percentile) exposure scenarios were considered. For the low scenario, 5th percentile value for measured concentration, quantity of air inhaled/dust ingestion, uptake factor, and biotransformation factor were used. The same was applicable for the intermediate and high scenarios, using median and 95th percentile values respectively. The fraction of time indoors and assumed body weight was the same for all three scenarios. Only exposure to 6 PFAS compounds was considered in the calculation; PFHxA, PFOA, PFNA, PFDA, PFHxS, and PFOS. This choice was based on three reasons: (i) only four PFAS (PFOA, PFNA, PFHxS, and PFOS) are included in the EU tolerable weekly intake (TWI) as explained in the discussion section of the paper; (ii) applicable serum concentrations available only contained a subset of target compounds (including PFOA, PFNA, PFDA, PFHxS, and PFOS); and (iii) PFHxA was included as air and dust concentrations were dominated by 6:2 FTOH which is a precursor of PFHxA, as discussed below.

Fraction uptake, F_{uptake}, values for PFOA and PFOS were previously estimated to be 0.66, 0.80, and 0.91 for low, intermediate, and high exposure scenarios respectively.[7] As done by Gebbink et al., 2015, and Vestergren et al., 2008, the same values were used for uptake of PFOS precursors (MeFOSE, EtFOSE, MeFOSA, EtFOSA), and for other PFAAs as there is a lack in data.[8,9] As also noted by Gebbink et al., 2015, bioavailbity in rats for diPAPs has been reported to be highly variable.[8] Moreover, some diPAPs, such as 10:2 diPAP, was not observed in the rats after dosing, but is known to be bioavailable to humans as it has been detected in human blood.[8] For this reason, Gebbink et al., 2015 assumed the uptake factors for diPAPs to be the same as those used for PFOA and PFOS (i.e. 0.66, 0.80, and 0.91 for low, intermediate, and high exposure scenarios respectively).[8] For that reason, the same assumption is also made in this study. Fraction uptake values used for the FTOH precursors were 0.27, 0.38, and 0.56 as reported by Vestergren et al., 2008.[9]

Biotransformation factors, $F_{biotransformation}$, were used as reported by Gebbink et al., 2015. In regards to PFOS precursors, there are reported factors of 0.095 (low), 0.20 (intermediate), and 0.32 (high) for the biotransformation of EtFOSE and FOSA to PFOS.[8] However, there is a lack of data available for other PFOS precursors, and so, as Gebbink et al., 2015 also reported, these values were used for the biotransformation factors of all PFOS precursors. While the biotransformation of FTOHs and diPAPs has been reported to produce several terminal PFCAs in in vivo and in vitro studies, only biotransformation to the promidently produced PFCA was considered, as done by Gebbink et al., 2015. For example, 8:2 FTOH or 8:2 diPAP predominantly produced PFOA and only to a minor extent, other PFCAs.[10,11] For that reason, this study considered 6:2 FTOH and 6:2 diPAP indirect exposure to PFHxA, 8:2 FTOH and 8:2 diPAP indirect

exposure to PFOA, and 10:2 FTOH indirect exposure to PFDA. For FTOHs, Vestergren et al., 2008, discusses the variablity across reported biotransformation factors and compiles the literature data to represent the variation, giving values of 0.0002, 0.005, and 0.017 for low, intermediate, and high exposure scenarios respectively.[9] For diPAPs, there is very limited literature on biotransformation factors but values have been determined for rats and shown to be chain-length dependent.[8] Gebbink et al., 2015, reported biotransformation factors of 0.01 for diPAPs with shorter chain length (6:2 diPAP) and 0.1 for diPAPs with longer chain length (8:2 diPAP), therefore these vaules were use for an intermediate scenario.[8] Also as Gebbink et al., 2015, biotransformation factors of 10 times lower and higher than the intermediate values were used for the low and high exposure scenarios.[8]

When considering indirect exposure to PFAAs, the EDI calculation uses a 1:1 molar basis for conversion of precursors, such as FTOHs, into PFAAs. In the case of diPAPs, the molar concentration is multiplied by two, as the diPAP has twice the same chain length (for example, 6:2 diPAP).[8]

All values used can be found in table format in the SI excel, Table S13.

EDI results from additional calculations can be found in the SI excel, Table S15 (under *Additional Calculations*). These numbers represent i) estimated daily direct intake via dust for all ionic compounds measured in dust (as shown in SI excel, Table S9b), not just ionic compounds discussed in the main text for comparison against the EU TWI, and ii) estimated daily direct intake for volatile PFAS via both air and dust. For direct exposure to volatile PFAS, the indirect PFAA intake equation (equation 3) was used without biotransformation factors.

Predicted Serum Concentration Calculations

The predicted serum concentration is a sum of the predicted serum concentration from dust (both through direct and indirect exposure) and from air (from indirect exposure) (equation S1).

$$Total\ predicted\ serum\ concentration = C_{serum\ from\ dust} + C_{serum\ from\ air} \quad \text{(eq S1)}$$

where $C_{serum\ from\ dust}$ and $C_{serum\ from\ air}$ are calculated using equation S2 and equation S3 below.

$$C_{serum\ from\ dust} = \left(\frac{C_{PFASdust} \times q_{dust} \times f_{time-in} \times F_{uptake} \times F_{biotransformation} \times T_{1/2}}{BW \times V_D \times \ln(2)}\right) \quad \text{(eq S2)}$$

$$C_{serum\ from\ air} = \left(\frac{C_{PFASair} \times q_{air} \times f_{time-in} \times F_{uptake} \times F_{biotransformation} \times T_{1/2}}{BW \times V_D \times \ln(2)}\right) \quad \text{(eq S3)}$$

where

$C_{PFASdust}$ and $C_{PFASair}$ are the concentrations of measured PFAS in air (ng m^{-3}) and dust (ng g^{-1});

$T_{1/2}$ is the half-life of the PFAA in the body (days); and

V_D is the volume of distribution (mL/kg) of the PFAA in the body.

Inhalation rates, ingestion rates, fraction of time indoors, uptake factors, biotransformation factors (used in the case of precursor compounds), and bodyweight were the same values as those used in the EDI calculations. Half-life and volume of distribution parameters were taken from previously published work.[12]

For the predicted serum calculations, the same concept of low, intermediate, and high exposure scenarios as used in the EDI calculations were employed, as described above. All values used for half-lives ($T_{1/2}$) and volume of distribution (V_D) parameters were used in previous studies to determine source contribtuions to blood serum concentrations.[12] Half-lives for PFOA and PFOS were taken from Xu et al., 2020.[13] Half-lives for PFNA and PFDA were taken from Hu et al., 2019, and Zhang et al., 2013, respectively.[14,15] Volume of distribution values were provided by Thomspon et al., 2010, for PFOA and PFOS, and by Ohmori et al., 2003, for PFNA and PFDA.[16,17] In the case of precursors, $T_{1/2}$ and V_D was determined to be that of their terminal compound. For example, in the case of 8:2 FTOH, $T_{1/2}$ and V_D of PFOA was used. It was also assumed that, like in the EDI calculations, the concentration of a precursor contributed to the blood serum concentration associated with it’s terminal PFAA. For the complete dataset of individual compound’s parameters used, see SI Table S17.

PFAS Concentrations in Air and Dust Samples

In the air samples, 6 neutral target PFAS were detected in at least one home, with fluorotelomer alcohols (FTOHs) dominating in both concentration and detection frequency (Figure 1). For example, 6:2 FTOH was detected in all but one home, ranging from 1.54 to 40.7 ng/m^3 when detected, with a median of 4.75 ng/m^3, followed by 8:2 FTOH, detected in all homes ranging from 0.785 to 15.1 ng/m^3, and 10:2 FTOH, detected in all but one home ranging from 0.259 to 4.19 ng/m^3 when detected (Table S8b). N-Ethyl perfluorooctane sulfonamido ethanol (EtFOSE) was detected in around half of the homes, ranging from 0.061 to 0.085 ng/m^3 when

detected. Less commonly detected in air were the fluorotelomer acrylates, 8:2 FTAcr and 10:2 FTAcr, which ranged from 0.491 to 0.575 ng/m^3 and 0.381 to 0.436 ng/m^3, respectively, when detected (Table S8b).

In dust samples, 8 neutral target PFAS were detected in at least one home (Table S9d). Like air samples, FTOHs had the highest measured concentrations, with 6:2 FTOH ranging from 17.6 to 94.3 ng/g dust, when detected, and a median of 26.6 ng/g dust, but N-methyl perfluorooctane sulfonamide (MeFOSA) and N-ethyl perfluorooctane sulfonamide (EtFOSA) were most frequently detected (Table S9d). Other compounds detected in over half of the homes included 8:2 FTOH (ranging from 13.5 to 93.3 ng/g dust when detected), 10:2 FTAcr (ranging from 9.73 to 14.5 ng/g dust when detected), and N-methyl perfluorooctanesulfonamido ethanol (MeFOSE) (ranging from 15.3 to 23.2 ng/g dust when detected) (Table S9d). 10:2 FTOH and EtFOSE were the least frequently detected in dust, with ranges from 14.9 to 49.2 ng/g dust and 15.3 to 19.5 ng/g dust, when detected, respectively (Table S9d).

Ionic PFAS were only measured in dust samples, with 22 target compounds detected in at least one home (see SI Table S9b for full list of detected compounds). PFCAs (C4-C14) were the most frequently detected, found in 100% homes above detection limits, followed by the disubstituted polyfluoroalkyl phosphates (diPAPs), PFOS, and perfluorodecanesulfonic acid (PFDS), which were detected in at least 85% of homes (Table S9b). Of the PFAAs, PFDS was detected at the highest concentration ranging from 0.949 to 12.2 ng/g dust when detected, with a median of 3.20 ng/g dust, followed by PFHxA, ranging from 0.522 to 14.3 ng/g dust, PFOS, ranging from 0.310 to 24.2 ng/g dust when detected, and perfluorooctanoic acid (PFOA), ranging from 0.493 to 16.7 ng/g dust (Table S9b). Detected at lower concentrations were PFNA, ranging

from 0.145 to 2.61 ng/g dust, PFDA, ranging from 0.207 to 9.25 ng/g dust, and PFHxS, ranging from 0.264 to 1.51 ng/g dust when detected (Table S9b). Of all measured ionic compounds, 6:2 diPAP was detected at the highest concentration, ranging from 0.280 to 48.8 ng/g dust when detected (Table S9d). 8:2 diPAP was frequently detected and seen at concentrations similar to PFDA, ranging from 0.120 to 8.10 ng/g dust when detected (Table S9d).

Air-dust correlations

Inverse correlations were present between compounds in measured in dust. For example, perfluoropentanesulfonic acid (PFPeS), perfluorotridecanoic acid (PFTrDA), and FTOHs were all negatively correlated with both MeFOSA and EtFOSA (Figure 3). The strong negative correlation between PFPeS and both MeFOSA and EtFOSA ($R^2 > 0.5$) is likely due to the low detection frequency of PFPeS across the 40 homes (SI, Table S9b and Table S11). However, while PFTrDA, 6:2 FTOH, and 8:2 FTOH were found above detection limits in more than 70 % homes, the corresponding correlations are weak ($0.1 < R^2 < 0.4$) (SI, Table S11).

Air-dust Partitioning

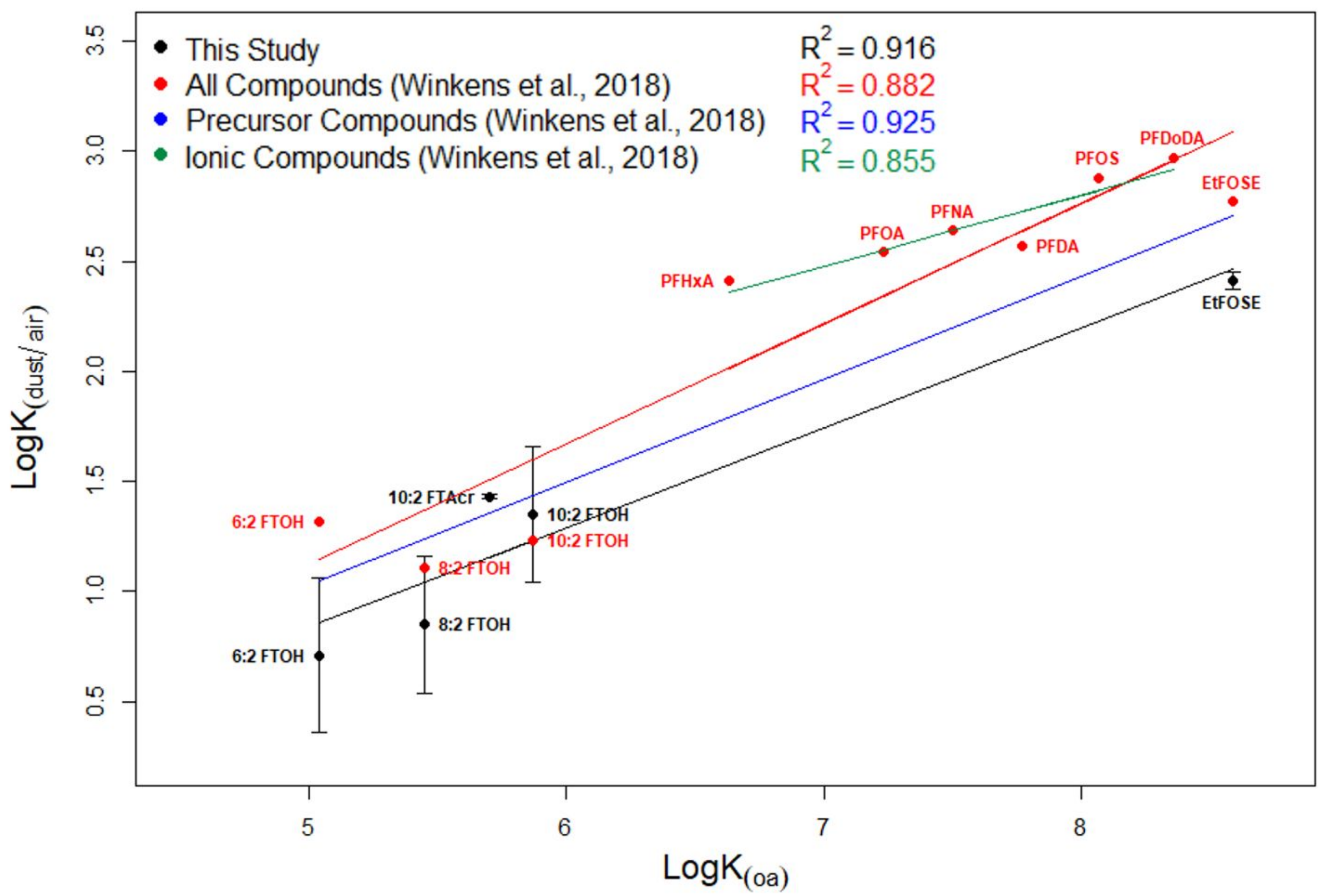

Figure S2. In black, $LogK_{dust\text{-}air}$ vs. $LogK_{oa}$ for neutral compounds detected in this study; y = 0.45 (±0.08)x - 1.43; (n = 40, median values); uncertainty calculated as one standard deviation; $LogK_{oa}$ values determined by Dreyer et al., 2009[19] and Wang et al., 2011[20]. In red, Winkens et al., 2018[18]; y = 0.55x – 1.61; (n = 55, median values). In blue and green, Winkens et al., 2018, data trendlines separated into neutral (y = 0.47x – 1.31) and ionic (y = 0.32x – 0.24) compounds respectively.

Contribution to total exposure

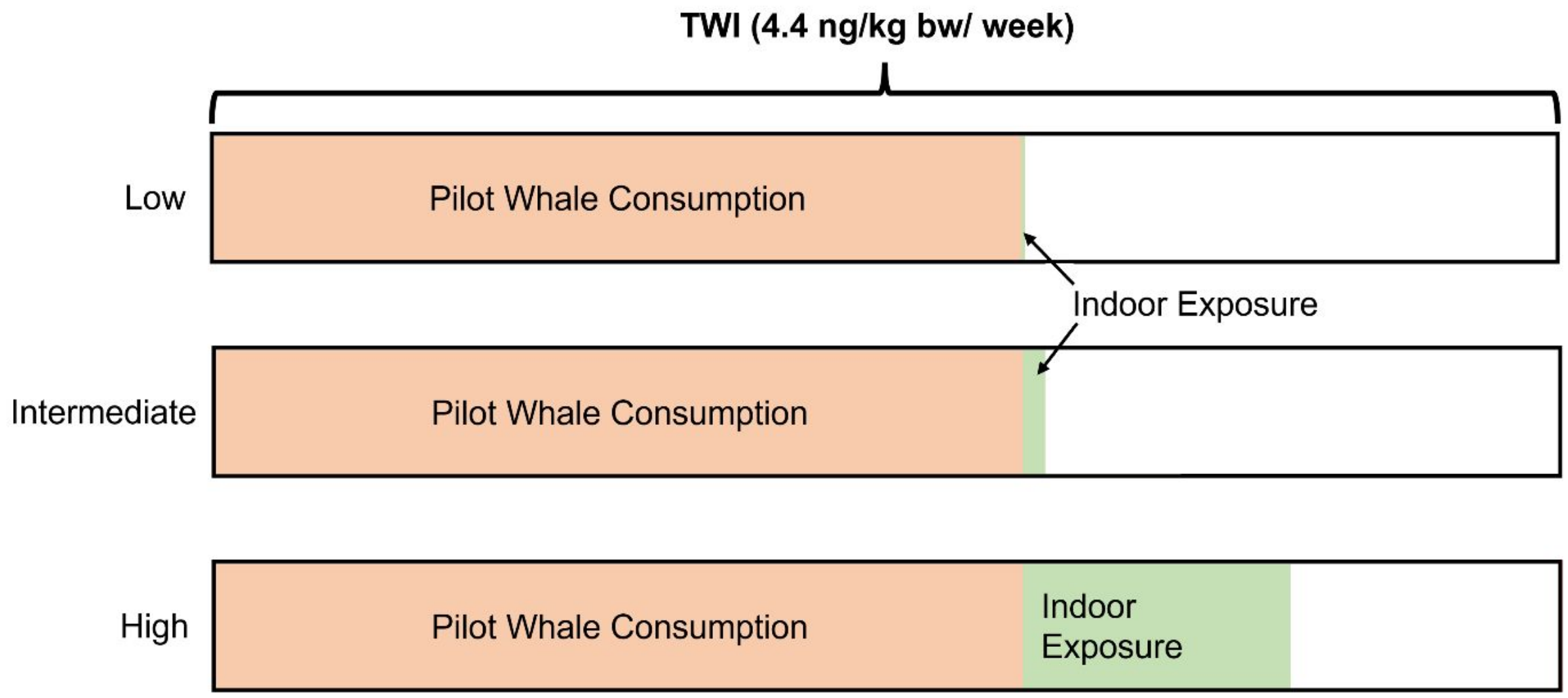

Figure S3. EDI from indoor exposure for children in low, intermediate, and high exposure scenarios, in contrast to average (when consumed) pilot whale consumption and the EU TWI.

Predicted Serum Concentrations

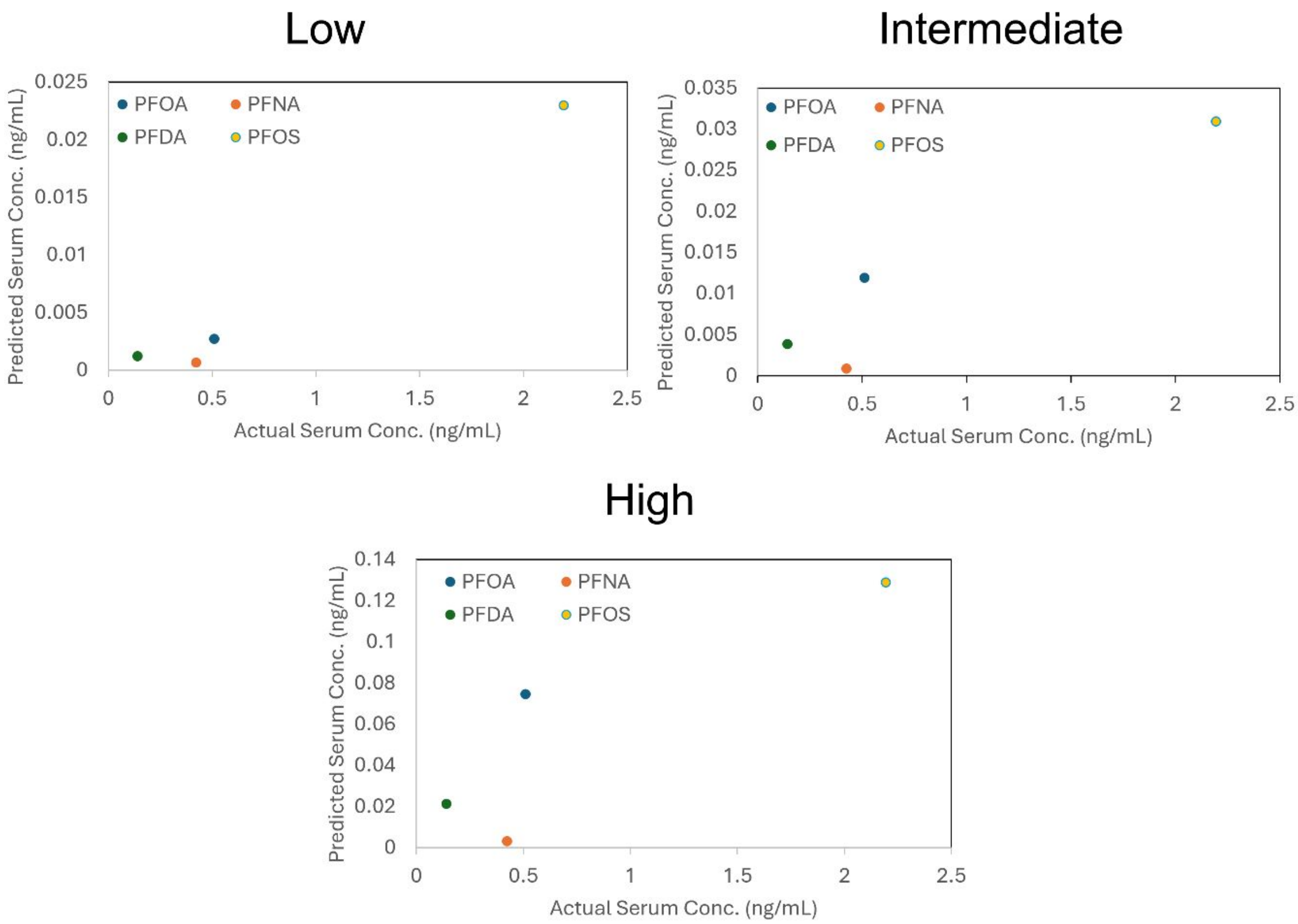

Figure S4. Plots showing the predicted serum concentration versus actual serum concentration for low, intermediate, and high exposure scenarios.

References

(1) Wu, Y.; Romanak, K.; Bruton, T.; Blum, A.; Venier, M. Per- and Polyfluoroalkyl Substances in Paired Dust and Carpets from Childcare Centers. *Chemosphere* **2020**, *251* (xxxx), 126771. https://doi.org/10.1016/j.chemosphere.2020.126771.

(2) Karásková, P.; Venier, M.; Melymuk, L.; Bečanová, J.; Vojta, Š.; Prokeš, R.; Diamond, M. L.; Klánová, J. Perfluorinated Alkyl Substances (PFASs) in Household Dust in Central Europe and North America. *Environment International* **2016**, *94*, 315–324. https://doi.org/10.1016/j.envint.2016.05.031.

(3) Morales-McDevitt, M. E.; Becanova, J.; Blum, A.; Bruton, T. A.; Vojta, S.; Woodward, M.; Lohmann, R. The Air That We Breathe: Neutral and Volatile PFAS in Indoor Air. *Environ. Sci. Technol. Lett.* **2021**, *8* (10), 897–902. https://doi.org/10.1021/acs.estlett.1c00481.

(4) Cahuas, L.; Muensterman, D. J.; Kim-Fu, M. L.; Reardon, P. N.; Titaley, I. A.; Field, J. A. Paints: A Source of Volatile PFAS in Air–Potential Implications for Inhalation Exposure. *Environ. Sci. Technol.* **2022**, *56* (23), 17070–17079. https://doi.org/10.1021/acs.est.2c04864.
(5) Schlummer, M.; Gruber, L.; Fiedler, D.; Kizlauskas, M.; Müller, J. Detection of Fluorotelomer Alcohols in Indoor Environments and Their Relevance for Human Exposure. *Environment International* **2013**, *57–58*, 42–49. https://doi.org/10.1016/j.envint.2013.03.010.
(6) Pu, S.; McCord, J. P.; Bangma, J.; Sobus, J. R. Establishing Performance Metrics for Quantitative Non-Targeted Analysis: A Demonstration Using per- and Polyfluoroalkyl Substances. *Anal Bioanal Chem* **2024**, *416* (5), 1249–1267. https://doi.org/10.1007/s00216-023-05117-4.
(7) Trudel, D.; Horowitz, L.; Wormuth, M.; Scheringer, M.; Cousins, I. T.; Hungerbühler, K. Estimating Consumer Exposure to PFOS and PFOA. *Risk Analysis* **2008**, *28* (2), 251–269. https://doi.org/10.1111/j.1539-6924.2008.01017.x.
(8) Gebbink, W. A.; Berger, U.; Cousins, I. T. Estimating Human Exposure to PFOS Isomers and PFCA Homologues: The Relative Importance of Direct and Indirect (Precursor) Exposure. *Environment International* **2015**, *74*, 160–169. https://doi.org/10.1016/j.envint.2014.10.013.
(9) Vestergren, R.; Cousins, I. T.; Trudel, D.; Wormuth, M.; Scheringer, M. Estimating the Contribution of Precursor Compounds in Consumer Exposure to PFOS and PFOA. *Chemosphere* **2008**, *73* (10), 1617–1624. https://doi.org/10.1016/j.chemosphere.2008.08.011.
(10) D'eon, J. C.; Mabury, S. A. Exploring Indirect Sources of Human Exposure to Perfluoroalkyl Carboxylates (PFCAs): Evaluating Uptake, Elimination, and Biotransformation of Polyfluoroalkyl Phosphate Esters (PAPs) in the Rat. *Environ Health Perspect* **2011**, *119* (3), 344–350. https://doi.org/10.1289/ehp.1002409.
(11) Martin, J. W.; Mabury, S. A.; O'Brien, P. J. Metabolic Products and Pathways of Fluorotelomer Alcohols in Isolated Rat Hepatocytes. *Chemico-Biological Interactions* **2005**, *155* (3), 165–180. https://doi.org/10.1016/j.cbi.2005.06.007.
(12) Zheng, G.; Eick, S. M.; Salamova, A. Elevated Levels of Ultrashort- and Short-Chain Perfluoroalkyl Acids in US Homes and People. *Environ. Sci. Technol.* **2023**, *57* (42), 15782–15793. https://doi.org/10.1021/acs.est.2c06715.
(13) Xu, Y.; Fletcher, T.; Pineda, D.; Lindh, C. H.; Nilsson, C.; Glynn, A.; Vogs, C.; Norström, K.; Lilja, K.; Jakobsson, K.; Li, Y. Serum Half-Lives for Short- and Long-Chain Perfluoroalkyl Acids after Ceasing Exposure from Drinking Water Contaminated by Firefighting Foam. *Environ Health Perspect* **2020**, *128* (7), 077004. https://doi.org/10.1289/EHP6785.
(14) Hu, X. C.; Tokranov, A. K.; Liddie, J.; Zhang, X.; Grandjean, P.; Hart, J. E.; Laden, F.; Sun, Q.; Yeung, L. W. Y.; Sunderland, E. M. Tap Water Contributions to Plasma Concentrations of Poly- and Perfluoroalkyl Substances (PFAS) in a Nationwide Prospective Cohort of U.S. Women. *Environ Health Perspect* **2019**, *127* (6), 067006. https://doi.org/10.1289/EHP4093.
(15) Zhang, Y.; Beesoon, S.; Zhu, L.; Martin, J. W. Biomonitoring of Perfluoroalkyl Acids in Human Urine and Estimates of Biological Half-Life. *Environ. Sci. Technol.* **2013**, *47* (18), 10619–10627. https://doi.org/10.1021/es401905e.
(16) Thompson, J.; Lorber, M.; Toms, L.-M. L.; Kato, K.; Calafat, A. M.; Mueller, J. F. Use of Simple Pharmacokinetic Modeling to Characterize Exposure of Australians to

Perfluorooctanoic Acid and Perfluorooctane Sulfonic Acid. *Environment International* **2010**, *36* (4), 390–397. https://doi.org/10.1016/j.envint.2010.02.008.

(17) Ohmori, K.; Kudo, N.; Katayama, K.; Kawashima, Y. Comparison of the Toxicokinetics between Perfluorocarboxylic Acids with Different Carbon Chain Length. *Toxicology* **2003**, *184* (2–3), 135–140. https://doi.org/10.1016/S0300-483X(02)00573-5.

(18) Winkens, K.; Giovanoulis, G.; Koponen, J.; Vestergren, R.; Berger, U.; Karvonen, A. M.; Pekkanen, J.; Kiviranta, H.; Cousins, I. T. Perfluoroalkyl Acids and Their Precursors in Floor Dust of Children's Bedrooms – Implications for Indoor Exposure. *Environment International* **2018**, *119* (July), 493–502. https://doi.org/10.1016/j.envint.2018.06.009.

(19) Dreyer, A.; Weinberg, I.; Temme, C.; Ebinghaus, R. Polyfluorinated Compounds in the Atmosphere of the Atlantic and Southern Oceans: Evidence for a Global Distribution. *Environ. Sci. Technol.* **2009**, *43* (17), 6507–6514. https://doi.org/10.1021/es9010465.

(20) Wang, Z.; MacLeod, M.; Cousins, I. T.; Scheringer, M.; Hungerbühler, K. Using COSMOtherm to Predict Physicochemical Properties of Poly- and Perfluorinated Alkyl Substances (PFASs). *Environ. Chem.* **2011**, *8* (4), 389. https://doi.org/10.1071/EN10143.
